# Significance of Simple Steatosis: An Update on the Clinical and Molecular Evidence

**DOI:** 10.3390/cells9112458

**Published:** 2020-11-11

**Authors:** Guillermo Mazzolini, Jan-Peter Sowa, Catalina Atorrasagasti, Özlem Kücükoglu, Wing-Kin Syn, Ali Canbay

**Affiliations:** 1 Gene Therapy Laboratory, Instituto de Investigaciones en Medicina Traslacional (IIMT), Universidad Austral-CONICET, Pilar Buenos Aires B1629, Argentina; guillermomazzolinirizzo@gmail.com (G.M.); CAtorrasagasti@austral.edu.ar (C.A.); 2 Liver Unit, Hospital Universitario Austral, Universidad Austral, G4VP+C5 Pilar Centro, Buenos Aires Province, Argentinien Buenos Aires B1629AHJ, Argentina; 3 Department of Internal Medicine, University Hospital Knappschaftskrankenhaus Bochum, Ruhr University Bochum, 44892 Bochum, Germany; jan.sowa@rub.de; 4 Department of Gastroenterology, Hepatology, and Infectious Diseases, Otto-von-Guericke University Magdeburg, 39120 Magdeburg, Germany; kucukoglu.ozlem@gmail.com; 5 Section of Gastroenterology, Ralph H Johnson VA Medical Center, Charleston, SC 29401, USA; synw@musc.edu; 6 Division of Gastroenterology and Hepatology, Medical University of South Carolina, Charleston, SC 29425, USA; 7 Department of Physiology, Faculty of Medicine and Nursing, University of the Basque Country, UPV/EHU, 48940 Leioa, Spain

**Keywords:** non-alcoholic fatty liver disease, non-alcoholic steatohepatitis, benign condition, disease progression, cardiovascular risk

## Abstract

Non-alcoholic fatty liver disease (NAFLD) is defined clinicopathologically by the accumulation of lipids in >5% of hepatocytes and the exclusion of secondary causes of fat accumulation. NAFLD encompasses a wide spectrum of liver damage, extending from simple steatosis or non-alcoholic fatty liver (NAFL) to non-alcoholic steatohepatitis (NASH)—the latter is characterized by inflammation and hepatocyte ballooning degeneration, in addition to the steatosis, with or without fibrosis. NAFLD is now the most common cause of chronic liver disease in Western countries and affects around one quarter of the general population. It is a multisystem disorder, which is associated with an increased risk of type 2 diabetes mellitus as well as liver- and cardiovascular-related mortality. Although earlier studies had suggested that NAFL is benign (i.e., non-progressive), cumulative evidence challenges this dogma, and recent data suggest that nearly 25% of those with NAFL may develop fibrosis. Importantly, NAFLD patients are more susceptible to the toxic effects of alcohol, drugs, and other insults to the liver. This is likely due to the functional impairment of steatotic hepatocytes, which is virtually undetectable by current clinical tests. This review provides an overview of the current evidence on the clinical significance of NAFL and discusses the molecular basis for NAFL development and progression.

## 1. Introduction

Nonalcoholic fatty liver disease (NAFLD) is now the leading cause of chronic liver disease worldwide [1], and represents a major cause of severe liver complications, including cirrhosis and hepatocellular carcinoma (HCC) [2]. The clinicopathological definition of NAFLD is the accumulation of fat in >5% of hepatocytes in the absence of secondary causes of fat accumulation, such as alcohol consumption >30 gr per day in men or >20 gr per day in women [3,4], or the use of drugs that can cause steatosis (e.g., amiodarone, steroids, tamoxifen, and others) [5]. This is important because alcoholic liver damage is indistinguishable from NAFLD by biopsy [6]. Unfortunately, both types of injury, either through excess calorie consumption or excess alcohol intake, often coexist in daily clinical practice.

The spectrum of NAFLD encompasses a range of conditions from simple steatosis or non-alcoholic fatty liver (NAFL) to non-alcoholic steatohepatitis (NASH), NASH fibrosis, NASH cirrhosis and NASH-associated HCC as end-stage disease [2,7]. NAFL or liver steatosis is defined by fat accumulation in liver tissue with only mild portal or lobular inflammation. Cellular injury, in the form of ballooning degeneration and lobular inflammation in addition to steatosis, have to be present for the diagnosis of NASH. Currently, the only reliable method to discern NAFL from NASH is histological evaluation after liver biopsy. A recent meta-analysis estimated that the overall global prevalence of NAFLD diagnosed by (ultrasound) imaging is around 25%, and the prevalence of NASH in the general population ranges between 1.5% and 6.45% [1]. By now, NAFLD has reached epidemic proportions across the globe, although there is a significant geographic variability [8]. Moreover, the prevalence of NAFLD is continuously increasing worldwide, with 1.2 million new cases projected in the USA annually until 2030 [9].

NAFLD is strongly associated with obesity, type 2 diabetes mellitus (T2DM), and metabolic syndrome (MS), a condition which is characterized by systemic inflammation and insulin resistance (IR), although it is not definitely clear whether NAFLD is simply the liver correlate of the MS or if NAFLD promotes development of MS and its component conditions (hypertension, T2DM, and cardiovascular disease) [10,11]. Critical to any clinical risk assessment for an individual with NAFLD is that the main cause of increased mortality is due to cardiovascular events [12].

The severity of liver fibrosis is the only histological finding that strongly predicts liver disease progression and liver-related mortality of NAFLD [13,14]. However, it remains unclear if NAFL and NASH are a continuum of disease stages and temporal transition, or if they are separate entities diverging in the early course of the disease, depending on genetic, epigenetic, and various environmental factors [15,16]. These diagnostic and prognostic uncertainties are confounded by sampling variability from liver biopsy and the inhomogeneous distribution of steatosis and inflammation in the liver tissue [17,18]. Moreover, there are few prospective longitudinal studies on NAFLD patients, with repeat liver biopsies to monitor disease development and careful follow-up. This lack of data limits our ability to fully discern the natural history of individual stages of NAFLD.

## 2. Can Patients with NAFL Progress to a More Advanced Disease Stage?

Without doubt, NASH and NASH fibrosis are associated with an increased risk of progression to end-stage hepatic disease and development or worsening of a variety of extra-hepatic metabolic complications [19]. In contrast major uncertainties remain if simple steatosis is truly a benign liver condition. As mentioned above, more data on prospective disease monitoring by (multiple) repeat biopsies are urgently required for a better understanding of the natural course of NAFLD. Reasons for currently open questions and discrepancies between published reports include the mis-diagnosis of liver disease, where patients with cryptogenic cirrhosis actually had NASH-related cirrhosis [20,21], variable study duration and follow-up [22,23], as well as sampling variability from liver biopsy [17]. In the following section, we try to compile data on the one hand suggesting that NAFL or simple steatosis is a benign condition (Table 1), and on the other hand, data suggesting that NAFL confers a substantial risk of disease progression and the development of comorbidities (Table 2).

### 2.1. Evidence in Favor of the Good Prognosis of NAFL Patients

Several studies have argued against the possibility that simple steatosis can progress to fibrosis progression and is associated with poor clinical outcome [24]. In 12 patients with bland steatosis (no inflammation at all), a repeat liver biopsy was performed after 7.6–16 years, which did not show progression to cirrhosis and patients had no serious liver complications [37]. Another study from Younossi et al. demonstrated that only the presence of fibrosis of any degree remained an independent predictor of liver-related mortality in the multivariate analysis [25]. In a large study (*n* = 646 patients) with a mean follow up of 20 years (0–40 years range), Hagstrom et al. showed that fibrosis stage is directly associated with liver-related mortality and overall survival [26]. A systematic review including 10 longitudinal NASH biopsy studies comprising a total of 221 patients described that age and portal inflammation on initial biopsy are independent predictors of progression to advanced fibrosis in patients with NASH [27]. In a study comprising 728 adults and 205 children from the Nonalcoholic Steatohepatitis Clinical Research Network increased portal chronic inflammation was associated with progressive NAFLD in both adults and children [38]. One hallmark study of Ekstedt et al. analyzed survival and causes of death in a cohort study of 129 patients diagnosed with biopsy-proven NAFLD in comparison with a matched reference population. From the initial cohort, 88 patients were reevaluated, and in 68, a repeat liver biopsy could be performed. The authors observed that the overall survival of NAFLD patients was significantly lower than that of the reference population. When separating this population in histologically confirmed NASH or steatosis with no or mild inflammation survival was significantly reduced only in NASH patients [28]. It is important to recognize that this study was limited by heterogeneity of the patient population and the low number of patients included. A follow-up study on this important work by the same group focused on different causes of mortality [14]. The core finding of this work was that a fibrosis stage of 3 or 4, independent of NAS/presence of NASH, was associated with an increased risk for overall, cardiovascular and liver related mortality. NASH without significant fibrosis (stages 0–2) was associated only with an increased mortality risk from HCC. Patients with a NAS < 5 (NAFL or borderline NASH) with fibrosis stages 0–2 exhibit no significantly increased mortality risk compared to the control population. Of note, the proportion of patients with stage 3–4 fibrosis was similar in patients with NAS < 5 (5%) and in patients with NAS ≥ 5 (5%). A limitation of this study was that the reference cohort was selected from population data, without any information on BMI, factors of the metabolic syndrome or hepatic steatosis.

Two studies by Dam-Larsen et al. on individuals with histologically confirmed steatosis/fatty liver without inflammation or fibrosis compared those with NAFLD (170) to patients with alcoholic liver disease (AFLD; 246). In this comparison it was observed that the prognosis in patients with NAFLD was good as only 1.2% progressed to cirrhosis compared to 22% of AFLD [29,30]; 48 (28%) of NAFLD patients and 188 (76%) of AFLD patients died during the follow up period (median 12.8 years). Cardiovascular events (37%) and extrahepatic cancer (17%) were the leading causes in NAFLD, and cardiovascular events (20%) followed by cirrhosis (17%) were the main causes of death in AFLD. Central limitations of this study was that histological assessment was limited to steatosis and fibrosis and that BMI was only available for 35% of AFLD patients. 

Angulo et al. performed a retrospective longitudinal analysis of 619 patients with NAFLD at centers in the United States, Europe, and Thailand over a period between 1975 and 2005 [13]. The central finding was that a total of 193 patients (33.2%) died or underwent liver transplantation. In multivariable analysis, only patients with fibrosis had shorter overall survival in comparison with patients without fibrosis. Limitations of the study included the low number of patients with NASH but without fibrosis, and that the mortality in the control group without NASH and fibrosis was significantly higher than previously reported.

The studies summarized herein (Table 1) show unmistakably that the main liver-related risk in NAFLD arises from advanced fibrosis and cirrhosis and that at least liver-related mortality is driven by NASH. However, this does not in itself imply that NAFL or steatosis without significant inflammation is without risk.

### 2.2. Evidence of Progression from Steatosis to Fibrosing-Steatohepatitis and Mortality in NAFL

Although the above described early studies demonstrated a different outcome in NAFLD patients based on the presence or absence of NASH, there is growing evidence challenging the dogma that NAFL is a non-progressive disease. Contrary to the claim that NAFL is a benign condition, several studies provide data objecting this statement (Table 2). In a prospective study of 52 patients with paired liver biopsies, it was observed that 28% of patients with a NAS below 5 had fibrosis progression. Moreover, 17 of 29 (58%) NAFL patients (NAS < 3) progressed to borderline NASH or NASH [31]. The conclusions of this study should be taken with caution because all patients in this study received lifestyle advice and metabolic monitoring, which might have altered the natural course of the disease and because the sample size in each subgroup is small. 

In another study data of 108 patients with NAFLD were analyzed, who had serial liver biopsies after a median of 6.6 years (1.3–22.6 years) [32]. The authors observed progression to NASH in 44% of patients with baseline NAFL. Of 27 NAFL patients 10 (37%) progressed to fibrosis, all 10 exhibited NASH in the follow-up biopsy. Of those NAFL patients with fibrosis progression 6 reached stage 3 fibrosis at follow-up biopsy. T2DM was present in 80% of NAFL patients with fibrosis progression but only in 25% of NAFL patients without progression. These findings are in line with a study on seventy patients with untreated NAFLD (25 with simple steatosis and 45 with NASH) with follow up biopsy after a mean duration of 3.7 years between 1998 and 2009 [16]. A substantial proportion (64%) of the 25 patients with simple steatosis on initial biopsy progressed to NASH. Severe ballooning was observed in eight and bridging fibrosis in six NAFL patients progressing to NASH. Of note ballooning progression and fibrosis was paralleled with a reduction in ALT. This finding supports the notion that classic liver enzymes (ALT and AST) are not useful for diagnosis, follow up, or disease monitoring in NAFLD [39]. Previous studies found high proportions of patients with liver enzymes in normal range with NASH or even advanced fibrosis [40,41,42]. Our own unpublished data of over 250 obese individuals with biopsy proven NAFLD resulted in significant differences of AST and ALT between patients with steatosis and patients with NASH. However, to allow discrimination of steatosis and NASH cut off concentrations around 19 U/l would have to be chosen with insufficient sensitivities and specificities (unpublished data). In a sample of outpatients in a gastroenterology unit, who were diagnosed with liver steatosis by ultrasound only, we found that cardiovascular and metabolic risk factors were significantly more common than in patients without liver steatosis [43]. This effect was independent of the underlying cause of the outpatient visit. This finding suggests that liver steatosis detectable by ultrasound may already be sufficient to indicate elevated cardiovascular risk, independent of definite NAFL or NASH diagnosis.

Mendelian randomization is an epidemiological method that avoids confounding factors (such as diet or physical activity) and is aimed to avoid reverse causation in observational studies [44]. Applying mendelian randomization Dongiovanni et al. analyzed data of more than 9000 individuals from the liver biopsy cohort (LBC), the Swedish Obese Subjects Study (SOS), and the population-based Dallas Heart Study (DHS) [33]. The risk alleles of PNPLA3, TM6SF2, GCKR and MBOAT7 and their effect on hepatic steatosis were evaluated in a polygenic risk score. The extent of hepatic steatosis was associated with liver damage, insulin resistance, dyslipidemia and hypertension. Strikingly the impact of genetic variants on liver damage was proportional to their effect on lipid accumulation in the liver. Within the LBC, hepatic fat and fibrosis were associated independently of inflammation, suggesting a causal effect of steatosis on fibrogenesis. Overall, the data of this meticulous analysis of a very large number of patients suggests that at least the genetically determined proportion of hepatic steatosis is likely to be a causal risk factor for the development of liver fibrosis and extent of liver damage in NAFLD. 

A systematic review and meta-analysis was performed by Singh et al. on 11 studies, including 411 patients with biopsy-proven NAFL and NASH who underwent paired liver biopsies at least one year apart. From six studies, the fibrosis progression rate (FBR) in 133 NAFL patients could be calculated, with 52 (39.1%) patients developing progressive fibrosis. From seven studies, FBR for 116 patients with NASH was derived, with 40 (34.5%) developing progressive fibrosis. The annual FBR was 0.07 stages in NAFL and 0.14 stages in NASH, resulting in a progression of one stage in 14.3 years for NAFL and in 7.1 years for NASH. It should be noted that while the rate of progression was slower in NAFL, the proportion of patients who actually exhibited fibrosis progression did not differ significantly between NAFL and NASH [34]. Limitations of this meta-analysis include the heterogeneity of the studies analyzed, and that seven of them enrolled less than 25 patients.

Adams et al. analyzed the natural history of 103 patients, who underwent serial liver biopsies with a mean interval between biopsies of 3.2 ± 3.0 years [35]. When excluding cirrhotic patients, who cannot further progress, the annual FBR in these patients was 0.09 ± 0.67, which corresponds to the overall FBR of NAFLD found by Singh et al. [34]. NASH was present in 96 (93%) of the 103 patients. Of the three patients with bland steatosis on initial biopsy, two developed NASH. All four patients with steatosis and nonspecific inflammation on initial biopsy developed NASH. The proportion of patients who progressed in fibrosis stage (overall 37%) did not differ significantly between NAFL (34.4%) and NASH (53.8%). The main determinant of fibrosis progression over time was the fibrosis stage on initial biopsy, with lower stages (0–2) at higher risk. Progressive inflammation, steatosis, and ballooning were not associated with fibrosis progression. Of note, progression of fibrosis stage was associated with a reduction in ALT and AST as well as severity of steatosis. The findings of this study are limited by the very low number of NAFL patients (9). 

In the above described study by Eksted et al. that demonstrated increased mortality only in NASH patients [28], a histological course was also analyzed in available repeat biopsies. Progressive fibrosis was observed in 29 of 70 (41%) of NAFLD patients. In patients with steatosis without fibrosis, 17 of 36 (47%) developed fibrosis. It should be noted that this study was performed in patients with elevated serum AST or ALT, which preselects patients with more severe disease course. Moreover, patients with NASH in this cohort were significantly older (54.5 ± 12.4 years) than those with steatosis, with or without unspecific inflammation (46.7 ± 12.3 years). The most recent analysis from a matched cohort study of all Swedish patients with biopsy confirmed NAFLD (10568 patients) found an increased risk of mortality for all histological stages [36]. Compared to age, sex, and location matched controls of the general population, the mortality risk was 1.71-times higher in simple steatosis, 2.14-times higher in non-fibrotic NASH, 2.44-times higher in non-cirrhotic fibrosis, and 3.79-times higher in cirrhosis. The NAFLD-associated excess mortality derived from extrahepatic cancer, cirrhosis, cardiovascular disease and hepatocellular carcinoma.

The results summarized above collectively support the concept that NAFL can progress to NASH with fibrosis, mainly in patients who have poor metabolic control. Some studies suggest that fibrosis can even develop or progress without a transition from NAFL to NASH. Overall roughly 30–40% of NAFL patients seem to exhibit progression of fibrosis in studies with sequential biopsies. In some cases, this progression occurred within a relatively short time frame. These observations should be taken with some caution. On the one hand, sampling variability between two liver biopsies due to the inhomogeneous distribution of inflammation and ballooning might lead to incorrect classification of a region with lower disease activity, and subsequently ‘disease progression’ upon second biopsy in a region of representative disease activity. On the other hand, we need to consider the possibility of selection bias in studies which examined only those with follow-up biopsies, as these are usually individuals most at risk and/or likely to progress [45]. However, a proportion of 30–40% with disease progression would imply a substantial variability or observer error in histological assessment.

Regarding mortality, there is no doubt that histologically proven NASH and in particular advanced fibrosis and cirrhosis are associated with a higher risk of overall and especially liver-related mortality. Though, NAFLD as a whole exhibits increased mortality rates compared to the general population. A recent analysis suggested that up to 29% of healthy controls may actually have NAFLD [46], masking a part of the impact of NAFLD on health and clinical outcomes. Some studies referred to above used population based controls without correction for BMI or metabolic morbidities to compare mortality to NAFLD or NAFL patients. This would probably underestimate the true effect of NAFLD on mortality, especially as non-cirrhotic stages and even compensated cirrhosis in NAFLD are still widely underdiagnosed in daily practice [47].

EASL Clinical Practice Guidelines indicate that NAFL patients without worsening metabolic risk factors should be monitored at 2–3-year intervals considering the low risk of progression for this group of patients [48], although this may be a too long an interval for patients with an underestimated risk of progression. Thus, it is imperative to identify reliable risk factors for progression and subsequently improve the surveillance of patients with NAFL at risk of progression. Currently the disease progression of any NAFLD patient cannot be excluded with certainty, and thus it is mandatory to improve the efficacy of surveillance to prevent as many liver-related and other metabolic complications as possible. 

## 3. Pathophysiological and Molecular Mechanisms of Non-Alcoholic Fatty Liver Disease

**Figure 1:**
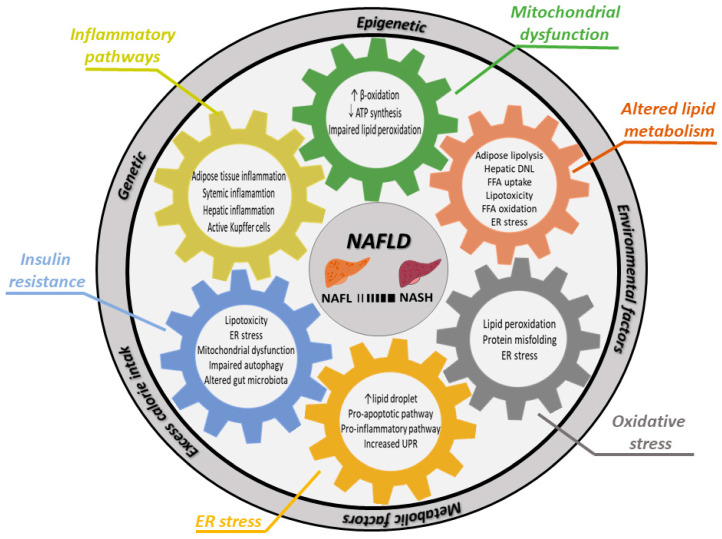
Factors contributing to development of NASH or progression of NAFL to NASH. NAFLD develops due to excess calorie intake, which leads to adipose tissue hypertrophy. Adipose tissue confronted with nutrient overload will increase lipolysis and change secretion of adipokines. This altered systemic situation of nutrients, lipids and adipokines, ultimately resulting in NAFLD, is modulated by genetic, epigenetic, environmental and metabolic factors (outer ring). Various molecular and cellular mechanisms (cogs) interact in development of NAFLD and NASH. Abbrevations: ATP: adenosine triphosphate; β-oxidation: beta oxidation of lipid components within mitochondria; DNL: de novo lipogenesis; ER stress: endoplasmatic reticulum stress; FFA: free fatty acids; UPR: unfolded protein response.

NAFLD is the consequence of excess calorie intake (from overnutrition and inactivity) and subsequent adipose tissue hypertrophy, with different combinations of genetic, epigenetic, environmental and metabolic factors interacting in development and influencing the severity of the disease. This complex genesis makes understanding of this disease a true challenge; though, it is now widely accepted that NAFLD is a multifactorial disease (Figure 1). The previous “two sequential hits pathogenesis” theory was based on insulin resistance (IR) and its stimulation of triglyceride (TG) deposits within the liver as initial hit, which is followed by a “second hit” induced by oxidative stress or ATP depletion, which further induces apoptosis, inflammation and fibrosis [49]. This theory has been replaced by the “multiple parallel hits theory”, in which several factors act in parallel and different combinations, sometimes synergistically, generating NAFLD [50]. This situation limits the development of pharmacological interventions, which could help the majority of patients, in contrast to HBV or HCV, where a single factor is causative for the disease. Patients with NAFLD seem to have individually different disease courses with varying contribution of IR and genetics [33] and some of them directly develop NASH while others (seem to) progress sequentially from NAFL to NASH [51]. Approximately 25–30% of patients with simple steatosis will develop NASH, and thus it is important to understand the molecular mechanisms involved and how to treat or prevent this complex disease. In this chapter of the review, we will discuss known key pathogenic factors involved in the genesis of NAFLD on a molecular level.

### 3.1. Genetic Factors Involved in NAFLD

By now it is clear that genetic factors contribute to the pathogenesis of NAFLD. Genome-wide association and gene expression profiling have identified genes with a number of polymorphisms associated with NAFLD development such as Patatin-like phospholipase domain containing 3 (PNPLA3), Transmembrane protein 6 superfamily member 2 (TM6SF2), GCKR, CETO, MBOAT7, MTTP, SOD2, GST, APOC3, IL-6, TNF-a, PPAR-a, just to mention a few [52]. In general, these genes or their products are involved in one or more of the following processes: lipid and glucose metabolism, insulin signaling pathways, oxidative stress (OS), detoxification, inflammatory pathways, and fibrogenesis. The two most studied and best characterized are PNPLA3 and TM6SF2 [52]. PNPLA3 is highly expressed in the liver and adipose tissue [53].

Although the function of PNPLA3 is not completely defined, it is considered to have lipogenic transacetylase activity. Presence of the PNPLA3 SNP rs738409 (I148M) promotes lipid storage in the liver, mainly because of a deficient glycerolipid hydrolase activity [53]. This effect seems to be more pronounced in normal-weight subjects than in overweight subjects [54,55]. The PNPLA3 variant allele rs738409 C>G has been associated with the risk and severity of NAFLD (inflammation, and progression to fibrosis) and even with HCC development [56]. PNPLA3 is a strong modifier of the natural history of NAFLD and can be considered as a potential target for therapy.

The TM6SF2 SNP rs58542926 C>T confers a significant genetic susceptibility for NAFLD and disease severity [57,58]; surprisingly, this mutation might confer cardiovascular protection [59]. The TM6SF2 variant has been associated with higher serum ALT and AST levels, and reduced plasma levels of TG and LDL-cholesterol [60]. In a mouse model, TM6SF2 silencing using an adeno-associated virus (AAV) vector increases hepatic TG levels and decreases plasma levels of TG, LDL- and HDL-cholesterols, demonstrating a key role of the TM6SF2 gene in hepatic TG secretion regulation [57]. 

Although genetic testing in NAFLD currently remains in the realm of research, it is likely that this could soon be in routine clinical practice, either as a prognostic indicator, or to risk stratify individuals for treatment. For example, individuals who are carriers of one or both alleles may be more at risk of progressive liver disease and therefore, could be targeted for more intensive surveillance, and/or receive therapy. However, it must be clear that the presence of the above described SNPs increases the risk to develop NAFLD or for a more severe course, but do not inevitably result in NASH.

### 3.2. Insulin Resistance

IR is one of the first metabolic changes occurring in overweight and obese individuals, associated with the systemic inflammatory state induced by obesity, often leading to more severe metabolic consequences. Adipose tissue inflammation and an altered adipokine profile promote peripheral IR. Hepatic IR with continued nutrient excess leads to lipotoxicity, ER stress, mitochondrial dysfunction, impaired autophagy, altered gut microbiota, which all promote progression from NAFL to NASH [50].

The detailed molecular mechanisms of IR that promote NAFLD development remain unclear. What is known is that under a chronic over-nutrition state and adipose tissue inflammation, an altered adipokine profile promotes a systemic inflammatory state, which further induces peripheral IR [61,62]. Adipose tissue IR promotes lipolysis, thus leading to an increase in circulating free fatty acids (FFA), which are then taken up by the liver for lipogenesis. In addition, gluconeogenesis and de novo lipogenesis (DNL) are stimulated [63]. In general, the transition from NAFL to NASH occurs in the presence of IR, however, the triggering events and molecular mechanisms regulating disease progression remain poorly understood. 

### 3.3. Lipid Metabolism and Lipotoxicity

Excessive hepatic accumulation of fatty acids constitutes the initial step of steatosis. In NAFLD patients, 59% of FFA are derived from deregulated lipolysis in adipose tissue, while the remaining 26% come from hepatic DNL, and only 15% originate from the diet [64]. Patients with high liver fat content exhibit at least threefold higher levels of DNL when compared with individuals with low liver fat content despite similar levels of FFA flux from the adipose tissue; therefore, DNL is a major contributor to the development of steatosis, apart from lipolysis [65]. Activation of the transcription factors sterol regulatory binding protein-1c (SREBP-1c) and the carbohydrate response element-binding protein (ChREBP) are crucial steps for DNL and FFA uptake [66]. Dietary cholesterol, dietary saturated fatty acid or hyperinsulinemia activate SREBP1c; chREBP is activated by hyperglycemia [67]. SREBP1c and ChREBP transactivate genes involved in FFA metabolism, including those required for FFA uptake (e.g., FABP3, FABP4, CD36), DNL (e.g., FASN and ACC1) and triglycerides (TG) synthesis (e.g., GPAT). Nevertheless, lipid accumulation is not necessarily harmful. The type of lipid and not the amount of lipids seems to determine lipotoxicity in hepatocytes [68]. Both TG storage in lipid droplets or TG export via VLDL secretion appear to have a protective role for the liver. In fact, transgenic mice overexpressing diacylglycerol acyltransferase 2 (DGAT2), which catalyzes the final step of TG synthesis, developed hepatic steatosis with reduced necro-inflammation [69]. Not lipid accumulation per se, but lipid composition seems to be crucial for disease progression from NAFL to NASH. While some lipid species are lipotoxic, others may protect cells from lipotoxicity. In contrast to unsaturated FFA, saturated FFA are detrimental to cell viability [70]. Unsaturated FFA (e.g., oleic acid) induce TG formation, which results in a mechanism of defense against the pro-apoptotic stimuli of large loads of saturated FFA [71,72]. Several other lipid metabolites, such as diacylglycerol, lysophosphatidyl choline, free cholesterol, cholesterol ester, ceramide, and bile acids, also accumulate in hepatic cells, and they promote ER stress, which leads to the execution of pro-apoptotic pathways and stimulates pro-inflammatory signals that activate Kupffer cells and HSCs, and induce mitochondrial dysfunction and ROS [73,74,75,76]. Alterations in bile acid composition and excretion contribute additionally to liver injury in NAFLD [74,76,77]. 

### 3.4. ER Stress

Endoplasmic reticulum (ER) stress is the result of the accumulation of unfolded/misfolded proteins within the ER, which is enhanced by oxidative stress (OS) [78]. Under healthy physiological conditions, reactive oxygen species (ROS) are produced in hepatocytes (mainly within the mitochondria), i.e., by FFA oxidation. However, when ROS are generated in excess, are not eliminated, or converted (e.g., to a less toxic compound) they can initiate cell death [79]. Excessive ROS production leads to lipid peroxidation and protein misfolding due to oxidation of polyunsaturated fatty acids and sulfhydryl group proteins, respectively, which are common events in NAFLD [80]. ER stress stimulates lipid droplets accumulation in the liver in a SREBP-dependent process [81]. It has been demonstrated that SREBPs, transcriptional master-regulators for fatty acid and cholesterol synthesis, are upregulated by OS and ER stress leading to lipid accumulation generated by DNL, TG, and cholesterol deposits in the liver [82]. Conversely, increased hepatocyte lipid content can also initiate and perpetuate chronic ER stress [83]. In addition, OS and ER stress interfere with lipoprotein secretion from the liver [84]. In addition, the unfolded protein response (UPR) is linked to the activation of DNL pathways, further increasing steatosis [85]. Chronic activation of UPR also induces hepatocyte death and inflammation [86]. The interaction of increased lipid uptake, DNL, and reduced lipoprotein secretion result in a fatty liver. The continued generation of ROS, i.e., by sustained FFA oxidation, can promote progression of NAFL to NASH. 

Thus, the presence of OS and, consequently, ER stress are key players in the pathogenesis of NAFLD and progression to NASH. Importantly, ER stress can be pharmacologically targeted, e.g., by the use of antioxidants (vitamin E) aiming at reducing excessive ROS production.

### 3.5. Mitochondrial Dysfunction

Increased mitochondrial activity is crucial to protect hepatocytes from the deleterious effect of FFA deposition since FFAs are oxidized by mitochondrial β-oxidation [87,88]. NAFL induces PPAR-α, which promotes FFA delivery to the mitochondria via CPT-1. However, during NAFLD development enhanced mitochondrial FFA β-oxidation leads to OS due to exacerbated leakage of electrons from the electron transport chain. This disrupts the balance between FFA removal and ROS generation [89]. Elevated ROS concentrations in the mitochondrial environment due to excess FFA may further damage the organelle function establishing a vicious circle. Hepatic mitochondria are structurally and molecularly altered in NAFLD [90], including electron transport chain alterations, and reduced ATP synthesis, which further induces ER stress. Increased mitochondrial cholesterol deposits induce changes in membrane permeability, and mitochondrial dysfunction; both processes have been linked to the progression of NAFL to NASH [79]. When ROS are produced in excess, both mitochondrial and nuclear DNA are damaged, impairing the transcription of mitochondrial proteins involved in metabolism and organelle biogenesis [91,92], which further aggravates the situation. Taken together impaired mitochondrial lipid peroxidation under a continuous supply of FFA leads to a vicious circle of excess ROS generation, ER stress, lipid accumulation, and mitochondrial impairment or damage. Currently, there are no known agents to inhibit this vicious circle apart from drastically reducing the influx of additional FFA, i.e., radical lifestyle change.

## 4. NAFLD Patients Are More Susceptible to Liver Injury Generated by Drugs and Alcohol

As described above simple steatosis is still considered generally a benign disorder, unlike NASH. Apart from the above described direct liver related and cardiovascular consequences of NAFL and NASH, several studies have shown that NAFLD worsens most conditions that injure the liver, suggesting increased sensitivity to viral hepatitides, alcohol, and drugs [93]. It is generally accepted that NAFLD patients will be more likely to be injured than healthy individuals by the same drug and dose [94,95]. In addition, drug-induced liver injury (DILI) remains a major concern for investigators in NASH clinical trials, and there are no evidence-based recommendations for best practices related to DILI in NAFLD patients [96]. In fact, the US Drug Induced Liver Injury Network (DILIN) reported recently that DILI in patients with chronic liver disease (including hepatitis C and NAFLD) was associated with more frequent adverse events, including mortality [97]. 

Different predisposing factors inherent to patients are known to enhance the risk of DILI including NAFLD and obesity [98,99,100]. In a prospective study on 248 patients with either HCV or NAFLD, NAFLD patients had a 3.95-fold risk of DILI compared to HCV [98]. NAFLD is associated with obesity, diabetes, hyperlipidemia, and other chronic diseases that require long-term and multiple drug administration. Therefore, increasing the risk of hepatic adverse effects or toxicity [101]. As such, clinicians should be aware of the added risk of liver injury in patients with chronic liver diseases, especially in NAFLD [102,103,104]. Apparently patients with NAFLD and/or obesity are more likely to suffer from drug-induced acute liver injury. NAFLD patients are also at risk of progression from NAFL to NASH as a result of DILI [100], at least for some types of drugs (e.g., acetaminophen, halothane, methotrexaye, pentoxufylline, rosiglitazone, tamoxifen) [105].

The increased susceptibility for drug-induced acute liver damage could be caused by various mechanisms, separately or in combination, such as increased expression of CYP2E1, inflammation, and reduced mitochondria respiratory chain activity; these might lead to increased ROS production, decreased ATP synthesis and production of pro-inflammatory cytokines (e.g., TNF-α) [106]. In addition, drug-induced liver damage (more frequently by isoflurane, acetaminophen, losartan, ticlopidine, and omeprazole) could be aggravated by the generation of higher levels of toxic metabolites produced by increased activity of CYP isoforms including CYP2E1 in obese individuals with NAFLD [94,98,99,107,108]. It has been shown that, in human liver samples, steatosis was associated with decreased hepatic cytochrome P450 3A (CYP3A) activity, which seems to be related to the severity of hepatic steatosis [109]. However, the link between decreased CYP3A activity and DILI in NAFLD patients is not robust.

Caregivers should consider that chronic administration of some drugs can worsen pre-existing NAFLD. Chronic alcohol intake in particular is detrimental to the steatotic liver as it may worsen the clinical course of NAFLD [110,111,112,113]. One study demonstrated a 5.3-fold risk of mortality for obese males drinking 1–14 drinks per week and a 18.9-fold risk of mortality for obese males drinking 15 or more drinks per week compared to normal weight non-drinkers [111]. Indeed, both drugs and alcohol can accelerate the transition from NAFL to NASH by increasing DNL, limiting the excretion of VLDL, and reducing fatty acid oxidation. In parallel the expression of pro-inflammatory cytokines, ROS production, and ER stress are further increased and mitochondrial respiratory chain activity is reduced [94,105,113,114]. 

Taken together, it is likely that NAFLD predisposes an individual to more severe liver injury by factors causing acute injury of hepatocytes as drugs, viral hepatitides, and alcohol. It should be noted that many of the above-referenced studies do not histologically discern NAFL from NASH. Therefore, it cannot be excluded that NAFL or liver steatosis alone could already enhance the risk of liver injury and/or aggravate the severity of liver injury, albeit probably to a lower extent than NASH.

## 5. COVID-19 and NAFLD

Coronavirus disease 2019 (COVID-19) is caused by a pathogen named Severe Acute Respiratory Syndrome Coronavirus-2 (SARS-CoV-2) [115,116]. In general, liver injury in COVID-19 patients was frequent but mild, and a clear association between any underlying hepatic disease with the course of the SARS-CoV-2 infection was not established. Underlying pathophysiological mechanisms of liver injury in COVID-19 remain largely unknown. However, in a series of 202 consecutive patients admitted (from January through February) to two COVID-19-designated hospitals in China up to March 2020, 76 (37.2%) had NAFLD [117]. The authors observed that patients with NAFLD showed a higher risk of disease progression to a more severe disease, and more persistent viral shedding in comparison with non-NAFLD patients [117]. In line with this, the EASL and ESCMID recommend to patients with NAFLD or NASH, which in addition may suffer from hypertension, diabetes and obesity, to adhere to physical distancing because they are at risk of a severe course of COVID-19 [118,119]. Another important issue in the scenario of COVID-19 in patients with chronic liver diseases, which includes NAFLD or NASH, is related to the use of experimental drugs that have been tested (such as chloroquine, hydroxychloroquine, lopinavir/ritonavir, remdesivir, camostat, tocilizumab, emapalumab, anakinra, among many others) to mitigate the most severe complications. Although clinicians have to keep in mind potential side effects of these drugs, it is also recommended to consider these patients for early treatment as they are at risk of progression [119]. Altogether, efforts should be made to better understand the role of NAFLD in COVID-19, and to be alert to the consequences of COVID-19 in patients with NALFD. 

## 6. Concluding Remarks

In summary, cumulative data show that NAFLD (which includes NAFL and NASH) may progress to severe chronic liver disease. NAFLD is also a risk factor for the development of other metabolic diseases (diabetes, cardiovascular disease) and may aggravate any other underlying liver disease. As such, clinical providers will need to recognize that NAFL/liver steatosis is not necessarily benign. As there is currently no non-invasive marker that can accurately and reliably distinguish NAFL from NASH, providers will have to use their clinical judgement in association with available tools to identify individuals likely to progress. While it seems obvious that NASH confers a greater risk of liver-related and overall morbidity and mortality than NAFL, NAFL is not without risk. In particular, progressive fibrosis and the development of cardiovascular morbidity occur in a similar proportion of NAFL and NASH patients. In conclusion, any patient with NAFLD must be considered an at-risk patient for the progression of liver disease and for the development of components of metabolic syndrome.

## Figures and Tables

**Table 1:** Evidence in favor of the good prognosis of NAFL patients.

Paired Liver Biopsy	# of Patients	Endpoints	Type of Study	Observations	Reference #
yes	40	natural history	retrospective	no progression to cirrhosis or liver-related complications; low number of patients	[24]
no	132	cirrhosis outcome, overall mortality, liver-related mortality	retrospective	poor outcome only in patients with ballooning, Mallory hyaline or fibrosis	[12]
no	209	liver-related mortality	retrospective	only fibrosis as independent factor of liver-related mortality	[25]
no	646	liver-related mortality, overall survival	retrospective	only fibrosis associated with liver-related mortality and overall survival	[26]
yes	221	natural history of fibrosis progression; predictors of progression to F3 fibrosis	retrospective systematic review	age and inflammation are predictors of progression to advanced fibrosis	[27]
Yes (*n* = 60)	129	survival and cause of death	retrospective	survival is lower in NASH but not in simple steatosis; low number of patients; heterogeneity of patient population;	[28]
no	170 NAFLD 246 ALD	risk of cirrhosis development; risk of death	retrospective	patients with simple steatosis have similar survival to Danish population	[29]
no	547	potential risk factors (index biopsy) for survival and cirrhosis development	retrospective	long-term follow-up study of ref. #31	[30]
No	619	long-term prognostic relevance of histologic features	retrospective	only fibrosis showed decreased overall survival, low number of patients with NASH and without fibrosis	[13]

Abbreviations: NAFLD, non-alcoholic fatty liver disease; ALD, alcoholic liver disease.

**Table 2:** Evidence in favor of the poor prognosis of NAFL patients (5 out of 6 with paired liver biopsies).

Paired Liver Biopsy	# of Patients	Endpoints	Type of Study	Observations	Reference #
yes	52	disease progression	prospective longitudinal study	20–30% of patients with simple steatosis had fibrosis progression; patients received lifestyle advice and metabolic monitoring	[31]
yes	108	factors predicting progression on liver biopsy		progression to NASH in 44% of patients with baseline NAFL	[32]
yes	70	progression of simple steatosis and mild inflammation to NASH and fibrosis	retrospective	ballooning in 16 of the 25 patients with simple steatosis, and bridging fibrosis in 6	[16]
no	1515 (liver biopsy cohort)	hepatic fat accumulation has a causal role in determining liver damage and insulin resistance	mendelian randomization approach	long-term hepatic fat accumulation plays a causal role in the development of chronic liver disease	[33]
yes	411 NAFLD - 150 NAFL - 261 NASH	clinical risk factors associated with progression	systematic review and meta-analysis	Liver fibrosis progresses in NAFL and NASH	[34]
yes	103	histological course of patients with sequential liver biopsies	retrospective	2 out of 3 patients with steatosis develop NASH; 4 out of 4 patients with steatosis and mild inflammation develop NASH; low number of patients	[35]
no	10,568	mortality in NAFLD	retrospective matched cohort study	increased risk of mortality for all histological stages 1.71-times increased risk in NAFL	[36]

Abbreviations: NAFLD, non-alcoholic fatty liver disease; NAFL, non-alcoholic fatty liver; NASH, non-alcoholic steatohepatitis.
